# Anterior Segment Ischemia in a Young Myopic following Transposition Surgery

**DOI:** 10.4103/0974-9233.53372

**Published:** 2008

**Authors:** Mona H. Al Enezi, Adnan H. Al Wayel

**Affiliations:** From the Department of Ophthalmology, Mohammed Abdul Rahman Al-Bahar Eye Center, Ibn Sina Hospital, Kuwait

**Keywords:** myopia, extra-ocular muscle transposition surgery, anterior segment ischemia, steroids

## Abstract

A 35-year-old Kuwaiti lady, who is a known myope, developed severe anterior segment ischemia following extra-ocular muscle transposition surgery. The patient was treated with topical and systemic steroids. Her best corrected visual acuity after complete resolution of the inflammation was 20/40. She also developed pupillary mydriasis and anterior lens changes.

Significant loss of anterior segment blood supply occurs when more than two rectus muscles are detached from the eye during surgery. Anterior segment ischemia (ASI) after extra ocular muscle surgery is caused by inadequate perfusion of the iris and ciliary body owing to disruption of an excessive number of anterior ciliary blood vessels.

There is a collateral blood supply to the anterior segment which consists of an anterior episcleral arterial circle (incomplete intra-muscular arterial circle) and a major arterial circle. The major contribution to this comes from the anterior ciliary arteries and each rectus muscle has two arteries except for the lateral rectus, which has only one artery.^1^

Severe ASI following strabismus surgery occurs in 30:400,000 cases, mostly with transposition of the vertical recti.

Mild cases are common and not significant. However, acute symptoms and signs of ASI typically become evident within one to two days after surgery. In severe cases the signs include: chemosis; corneal edema; uveitis; lens opacities; hypotony with iris sector perfusion defects occurring in around 20 percent;^2^ chronic sequelae including correctopia; iris atrophy; cataract and, on rare occasions, phthisis bulbi.

Strabismus surgery on the vertical recti is more likely to cause ASI. Two recti-muscle surgery rarely causes anterior segment ischemia unless there is a risk factor such as micro-vascular disease, sickle cell disease, leukemia, thyroid eye disease, high myopia and previous scleral buckling or strabismus surgery,^3^ radiation therapy.^4^

Here we report a case of ASI following augmented transposition of the vertical recti muscles, as described by Hummelsheim, in a known myopic patient.

## Case Report

A 35-year old woman presented to the clinic with a history of right squint following a road traffic accident one year earlier. On examination her right eye's best corrected visual acuity was 20/30 and 20/25 in the left eye.

Ocular motility examination showed absent abduction of the right eye. ACT showed right esotropia of 30 PD at near and 35 PD at far without correction. Examination of the anterior segment, pupils and fundi were unremarkable. Intraocular pressures measured 16 mmHg in each eye. Retinoscopy was RE: -7/-1.25 axis 10, LE: -4.5/-1.00 axis170.

The patient underwent right medial rectus recession (7 mm from the insertion) and a split-tendon transposition of the right superior and inferior recti to the lateral rectus insertion (Hummelsheim procedure)^5^ through a peritomy incision.

On the first day post-operatively the patient developed right eye corneal edema and anterior chamber activity (flare + cells). The right pupil was semi-dilated and irregular, and ocular movements were restricted in all directions. The intraocular pressure was 8 in the right eye compared to 14 in the left eye.

Anterior segment ischemia was diagnosed and topical and systemic steroids were initiated on the second post-operative day. Following initiation of the treatment, she started to improve.

Intraocular pressure was normal at 14/14 in the right eye on the forth post-operative day and the patient was discharged home on an oral steroid tapered over a two-week period, together with topical steroids.

**Figure 1:**
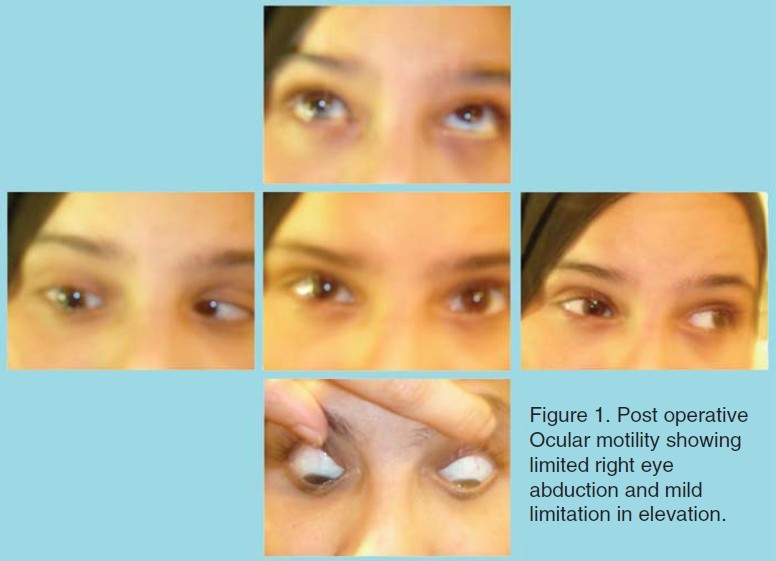
Post operative Ocular motility showing limited right eye abduction and mild limitation in elevation.

**Figure 2:**
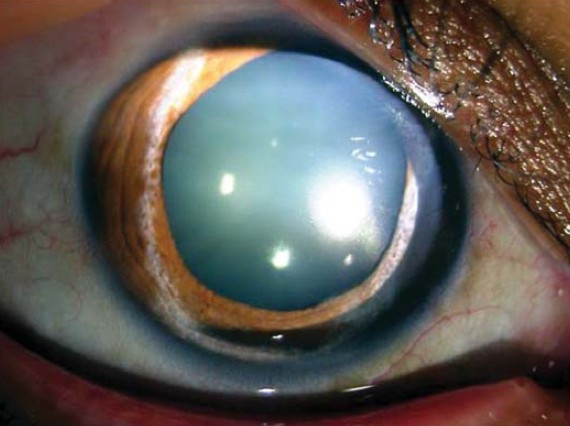
Right eye post operatively showing mydriasis, iris atrophy and anterior lens changes.

**Figure 3:**
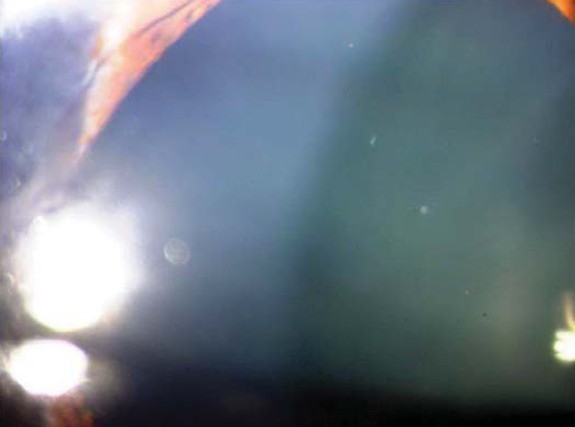
Anterior lens changes.

Sixteen months after surgery the patient had a BCVA of 20/40 in the right eye and 20/25 in the left eye. She was orthotropic in primary gaze and was able to abduct the right eye 10 degrees on right gaze.(Fig. 1) Anterior segment examination showed a right dilated non-reacting pupil,(Fig 2) iris atrophy, and anterior lens changes (Fig 3). Both fundoscopy and intraocular pressure were normal.

**Figure 4:**
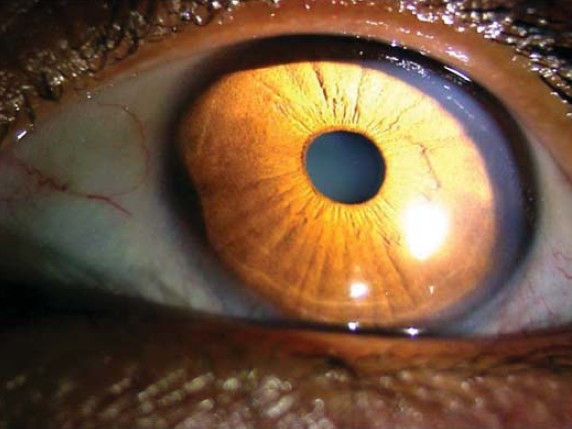
Normal left eye.

## Discussion

Our patient developed severe ASI on the first post-operative day. This patient presented with a known risk factor for ASI, as well as high myopia, which was unfortunately underestimated. If we had considered this risk factor a significant indicator for the possibility of developing severe ASI, we would probably have avoided this complication.

This patient would have benefited more from a staged procedure if the medial rectus recession had been done earlier, probably before three to four months, to allow for remodeling of the collateral circulation and an increased contribution from the long posterior ciliary arteries.^5^

A peritomy was performed on this patient.Limbal incisions are said to predispose a patient to more severe ischemia than fornix incisions by affecting the anterior episcleral arterial circle. Therefore a fornix conjunctival incision would have been a better option.^6^,^7^

Anterior segment circulation can be preserved by carefully dissecting the ciliary vessels from adjacent tissues before disinsertion and by then keeping the vessels intact during repositioning of the muscle.^6^ This was done but it did not protect the patient from ASI.

In conclusion, ASI can lead to a chronic sequel, though rare, and it must be kept in mind that in any strabismus surgery, especially in those patients of high risk, should not be undertaken without proper modification and surgical planning.
